# Nutrient digestion efficiency: a comparison between broiler chickens and growing pigs fed maize, barley and oats-based diets with an emphasis on starch

**DOI:** 10.1017/S0007114524003167

**Published:** 2025-01-28

**Authors:** Lucas S. Bassi, Marcin Hejdysz, Ewa Pruszyńska-Oszmałek, Paweł A. Kołodziejski, Aaron J. Cowieson, Sebastian A. Kaczmarek, Birger Svihus

**Affiliations:** 1 Department of Animal Nutrition and Feed Management, Poznań University of Life Sciences, Poznań 60-637, Poland; 2 Faculty of Biosciences, Norwegian University of Life Sciences, Ås 1433, Norway; 3 Department of Animal Physiology, Biochemistry and Biostructure, Poznań University of Life Sciences, Poznań 60-637, Poland; 4 DSM-Firmenich, Animal Nutrition and Health, Kaiseraugst 4303, Switzerland

**Keywords:** Broilers, Digestive physiology, Ingredient evaluation, Pig, Starch

## Abstract

We investigated the hypotheses that broilers and pigs have distinct starch digestion capacities and that different cereals could trigger diet–species interactions. Ten replicates of two broilers (14 d old) or one pig (50 d old) each were distributed into a 3 × 2 randomised factorial design with three pelleted diets (maize, barley or oat-based) and the two species. Nutritional composition was equal for both species. Diets were fed for 10 d, and then the pancreas and organs from the stomach region and small intestine were collected with contents. It was observed that both species were similarly efficient at digesting starch but differed in some digestive aspects. Broilers had higher ileal digestibility coefficients (*P* < 0·001) of DM (0·69) and crude protein (0·75) than pigs (0·66 and 0·67), presented a higher volume of particles < 0·1 mm in duodenal digesta (*P* < 0·001) and had a lower gizzard pH (3·68) than pig stomach (4·48; *P* < 0·05). Conversely, pigs had lower ileal viscosity (1·44 *v*. 2·77 cP; *P* < 0·05) and higher pancreatic lipase activity (27 *v*. 5·9 U/g of pancreas; *P* < 0·05) compared with broilers. In the jejunum, oat led to higher starch digestibility (0·96; *P* < 0·05) than maize and barley regardless of species. In the ileum, starch digestibility was higher for broilers fed oats (0·99) than broilers fed barley (0·94; *P* < 0·05), establishing that oats provided, in general, a superior starch availability. The results imply that starch utilisation capacity is more related to its dietary source than to the species to which it is fed.

Starch is an essential nutrient for non-ruminants and the primary source of energy derived from cereals, and its physiochemical properties have been thoroughly examined and discussed. Fundamentally, the amylose:amylopectin ratio, crystalline structure of the granules and concentration of fibre and NSP represent some of the main factors dictating the timeline of digestion and glucose release from starch granules^(1,2)^. These characteristics vary among the numerous cereals used in animal diets, such as maize, barley, oats and wheat^(3,4)^. Typically, starch from maize is recognised as highly available due to its low content of NSP relative to other cereals, depending on its endosperm type^(5)^. On the other hand, barley may contain up to 22 % fibre and higher levels of NSP than maize^(6)^, and oats are also rich in insoluble fibre coming from the hulls, although with higher protein and lower amylose contents than barley^(7)^.

Broilers exhibit a remarkable efficiency in starch digestion, often exceeding ileal starch digestibility coefficients above 0·95^(8–11)^. Pigs, akin to broiler chickens, are also recognised as efficient starch digesters^(12–14)^. However, situations where starch digestibility is low, that is, ≤ 0·9, can be experienced in both species^(15,16)^, with variations in starch digestion rates attributed to factors such as the presence or absence of exogenous enzymes, ingredient composition, feed processing, particle size, gelatinisation rates or the age of animals^(17)^. Nonetheless, a rapid starch digestion is considered an impressive feat for modern poultry birds given their relatively short mean retention time of feed in the small intestine (SI), ranging from 2–4 h^(18)^ when compared with pigs, that is, > 6 h^(19)^.

Considering how prominent broiler chickens and pigs are to animal farming and how relevant starch is to both, it is imperative to understand the differences and similarities related to starch digestion between these two dominant monogastric species. Currently, there is a paucity of data detailing the comparative digestive physiology of broilers and pigs. A review by Mcwhorter *et al.* ^(20)^ highlighted key aspects of the avian gut in comparison with mammals, and despite a relatively lower capacity of digestive organs and shorter digesta retention time, birds seem to have a greater villus amplification, leading to a higher mucosal surface area. Birds may also exhibit higher digestive enzyme activity or nutrient transport capacity that could compensate for their shorter tract. Moran^(21)^ further encompasses distinct features between the gastrointestinal tract of poultry and pigs, for example, the secretion of salivary *α*-amylase in pigs or varying viscosity in the SI. Furthermore, the mechanical grinding function of the gizzard^(22)^ and the extensive reflux of digesta through reverse peristalsis^(23)^ are examples of unique mechanisms of great importance to starch and overall nutrient digestion in poultry.

To the best of our knowledge, there are no comparative studies of starch digestibility between modern broilers and pigs fed the same diet. Understanding these differences in digestive mechanisms between species is not only academically intriguing but may also be used to optimise nutrition and feeding strategies, for example, through changes to diet composition, processing or inclusion of bioactive ingredients. This study aimed to explore physiological and dietary factors that influence nutrient digestion in broilers and pigs, with a specific emphasis on starch, built on the premise that both species have distinct starch digestion capacities. We hypothesised that broilers would present a superior capacity for starch digestion attributed to a higher amylase activity and the role of their anterior tract in grinding the feed to smaller particles. Moreover, different cereals were used to assess possible interactions with the species.

## Material and methods

All experimental procedures complied with the guidelines of the Local Ethical Committee for Experiments on Animals in Poznan (protocol no. 02/2024) regarding animal experimentation and animal care under study (European Union (EU) Directive 2010/63/EU for animal experiments).

### Animal husbandry - broilers

A total of sixty 1-d-old male Ross® 308 broiler chickens were acquired from a commercial hatchery (Dan Hatch Poland S.A., Stary Widzim 254, 64–200 Wolsztyn). At arrival, birds were group-housed on wood shaving litter in 1·2 × 0·8 m^2^ floor pens and fed a maize–soybean meal starter broiler diet (22·2 % crude protein (CP); 7·95 % crude fat; 0·96 % Ca; 0·48 % available P; 12·6 MJ metabolisable energy/kg in pelleted-crumbled form for 14 d. After 14 d of adaptation, birds were weighed, randomly selected and housed in pairs in thirty cages measuring 0·50 × 0·40 × 0·50 m (length × width × height), where they received pelleted experimental diets for 10 d. The average individual body weight at the start of the experiment was 424 g. Cages had wire-mesh floors and trays and were equipped with manual feeders and drinkers. Pelleted feed and water were offered on an *ad libitum* basis. Birds were exposed to light for 24 h per d in the first 7 d, followed by 18 h light:6 h darkness. The temperature was maintained at 32°C during the first week and gradually reduced to ∼23°C by the end of the third week. The daily routine included verification of temperature, feed and water supply and inspection of cages for dead and culled birds. No mortality was observed throughout the experiment.

### Animal husbandry - pigs

A total of thirty male 50-d-old growing pigs (Naima × (Pietrain × Duroc)) with an average body weight of 20·5 kg were individually housed in floor pens with straw, equipped with a nipple drinker and trough feeder with *ad libitum* access to water and feed. During a 6-d adaptation period, pigs were fed a pelleted maize–soybean meal diet (19·35 % CP; 5·05 % crude fat; 0·76 % Ca; 0·39 % available P; 12·5 MJ metabolisable energy/kg), which was gradually substituted until day 6 for the experimental diets, which were then fed for a period of 10 d. The daily routine included verification of temperature, feed and water supply and cleaning of pens. No mortality was observed throughout the experiment, but two pigs (one from maize and one from barley dietary treatment) showed symptoms of diarrhoea throughout the experiment (loss of weight, lack of appetite and soft faeces) and were therefore removed from sampling.

### Experimental design and diets

Animals were distributed into a 3 × 2 completely randomised factorial design, with three experimental diets (based on maize, barley or oats) and the two species (broilers and pigs), totalling six treatments with ten replicates of two broilers or one pig each. Three pelleted diets were produced, based on maize, barley or oats, and manufactured at the Experimental Station of the Department of Animal Nutrition and Feed Management Gorzyń/Miedzychód – Poland. Wheat was added to all diets as a complementary starch source. Maize and wheat were ground in a Skiold Disk mill (SK2500, Skiold Group) with a 1 mm disc distance, while barley and oats (both with hulls) were ground in a hammer mill (RG11 model, Zuptor) using a 3·4 mm screen. Minerals, amino acids, vitamins and fat were directly added along with the ground grains to a 100 kg horizontal mixer (model: Zuptor 100) with a 4 min mixing time and mixing speed of 27·4 rev/min. After mixing, all diets were pelleted using a Scorpion pellet press (BMG Pelleting Experts) equipped with a 22 kW engine and a 4 mm thick ring die with 3 mm diameter holes.

**Fig. 1.**
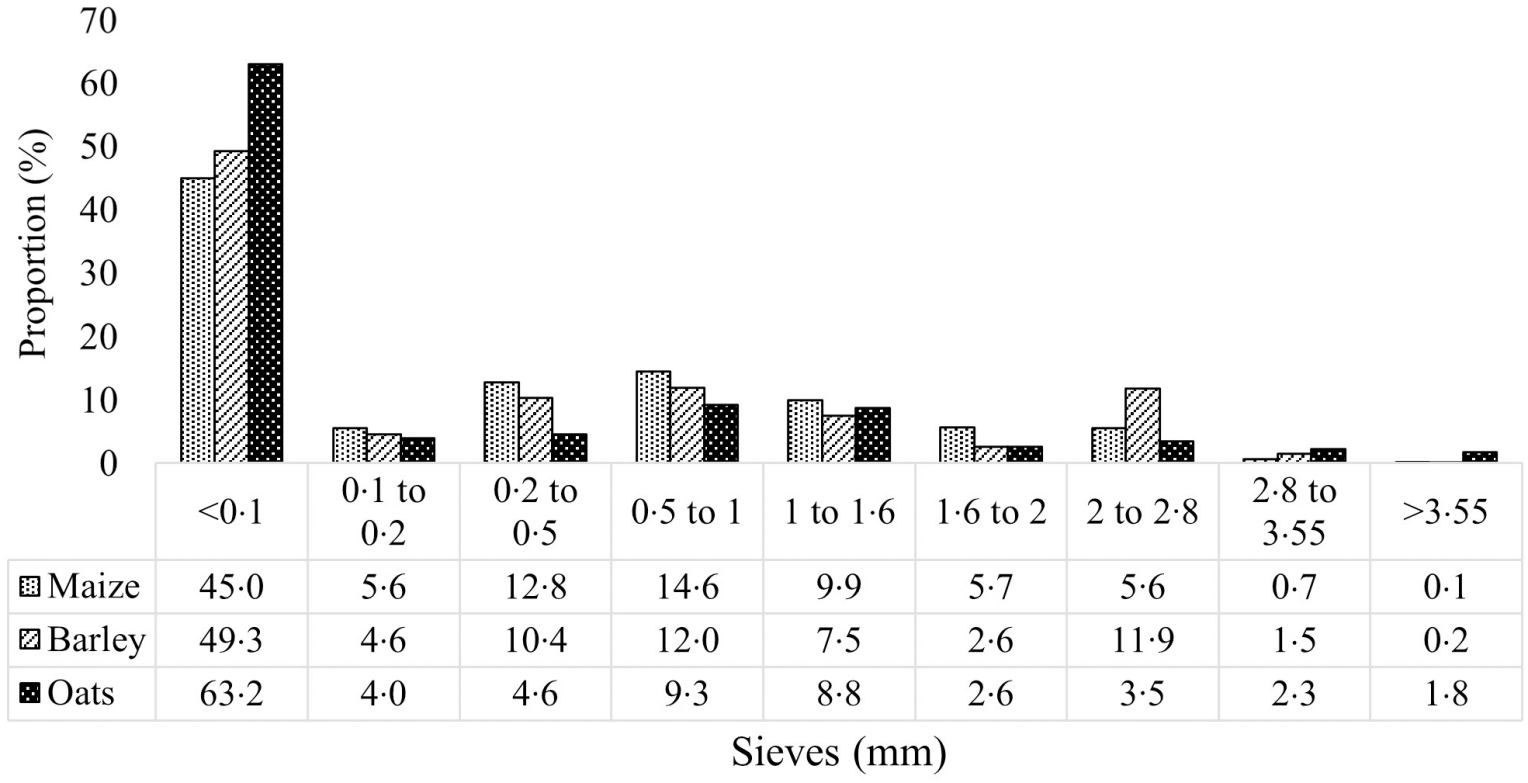
Particle size distribution (determined by wet sieving) of experimental maize-, barley- and oat-based pelleted diets.

Approximately 200 g of ground cereals (collected prior to pelleting) and pelleted diets (collected after cooling) were used in the determination of mean particle size through either dry or wet sieving^(24)^, which was then used in the calculation of geometrical mean diameter according to the American Society of Agricultural and Biological Engineers (method ANSI/ASAE S319·3 FEB03). The determined geometrical mean diameter of ground maize, wheat, barley and oats from dry sieving was 506, 500, 598 and 520 μm, respectively, and particle size distribution (PSD) of maize-, barley-, and oats-based pelleted diets from wet sieving is presented in Fig. 1. This particle size was decided upon based on recommended values for growing pigs at that age^(25,26)^.

Experimental diets (Table 1) were formulated to align with calculated average nutrient recommendations between finisher broiler chickens^(27)^ and growing pigs^(28)^. Vitamin and trace minerals levels were based on broiler chicken requirements, which were slightly above that of pigs, thereby ensuring that both species received sufficient quantities of micronutrients. The diets were not isonutrient due to inherent differences in the cereals. Maize, barley and oats varied significantly in their contents of CP, starch and non-nutrient fibre content (Table 2), making it impractical to include each cereal at the same level or to achieve similar starch content across diets. Instead, we focused on maintaining a consistent energy:CP ratio and a balanced proportion of starch coming from each of the main cereal sources, regardless of total starch content, hence the moderate difference in their inclusions at between 59 and 72 %. Using oats alone would greatly dilute dietary energy due to their high fibre concentration. To avoid compensating for this with an excessive fat inclusion, which would have dramatically altered the fat:starch ratio and jeopardised diet structure, wheat was added to the diet instead. Consequently, wheat was added to all three diets at moderately varying levels (9–12 %), which enabled the balance between starch proportions while maintaining energy:protein ratio. Maize, barley or oats were incorporated in varying proportions to attain a starch input of approximately 85 % from the investigated source and 15 % from wheat. Fat addition levels and to a smaller extent protein source levels were varied to uphold a fixed energy:CP ratio of approximately 64 MJ apparent metabolisable energy/kg CP. All diets contained 2000 units of phytase/kg of diet (Ronozyme HiPhos GT, dsm-firmenich) and an NSPase supplement (Ronozyme Multigrain, dsm-firmenich) containing endo-1,4-*β*-glucanase, endo-1,3 (4)-*β*-glucanase and endo-1,4-*β*-xylanase at 80, 70 and 270 units/kg of diet, respectively. Titanium dioxide (TiO_2_) was used as an indigestible marker in all diets.

**Table 1.** Ingredients and nutritional composition of experimental diets (as-fed basis)

Ingredient (g/kg)	Maize diet	Barley diet	Oat diet
Maize	593	–	–
Barley	–	617	–
Oat	–	–	717
Soybean meal	252	213	143
Wheat	120	105	90
Fish meal	10·0	10·0	10·0
Vitamin and mineral premix^*^	10·0	10·0	10·0
Calcium carbonate	3·20	3·50	3·70
Lysine HCl	3·10	3·30	4·50
Monocalcium phosphate	2·30	1·80	1·60
l-Valine	1·80	0·10	0·90
dl-Methionine	1·60	1·90	2·40
NaHCO_3_	1·40	3·00	3·30
Sodium chloride	0·80	0·00	–
Threonine	0·60	1·30	2·10
Rapeseed oil	–	30·0	10·0
l-Isoleucine	–	–	0·90
l-Tryptophan	–	–	0·20
Xylanase^†^	0·10	0·10	0·10
Phytase^‡^	0·10	0·10	0·10
Calculated nutrients in g/kg or otherwise noted (poultry/pigs)			
Metabolisable energy (MJ/kg)	12·6/12·8	12·4/12·6	10·6 /10·9
Crude protein	198	192	166
Energy:protein ratio (MJ/kg CP)	63·6/64·5	64·6/65·4	63·9/65·5
Available P	3·70	3·70	3·70
Total P	4·40	4·50	4·10
Na	7·70	7·70	7·70
K	7·70	7·30	6·50
Na	1·30	1·30	1·30
Chlorine	1·60	1·60	1·50
Digestible lysine	10·7/11·0	10·7 /10·8	10·6 /10·9
Digestible met+cys	7·10/6·70	7·10/7·30	7·10/7·40
Digestible tryptophan	1·80/2·00	1·80/2·10	1·80/2·00
Digestible threonine	6·80/6·50	6·80/6·80	6·80/6·90
Digestible arginine	10·7/11·0	10·0/10·5	8·90/8·60
Crude ash	45·1	49·3	48·5
Analysed nutrients in g/kg or otherwise noted			
Crude protein	210	208	179
Crude fat	29·5	50·0	46·6
Total starch	445	359	353
Proportion of starch from main cereal (%)	86·7	85·1	85·5
Proportion of starch from wheat (%)	13·3	14·9	14·5
Acid detergent fibre	36·1	55·9	90·5
Neutral detergent fibre	83·2	145	211
Soluble NSP	15·3	35·0	36·2
Insoluble NSP	69·0	95·4	165

* Provided per kg diet: mcg: retinol 3350, cholecalciferol 62·5; mg: vit. E 80, menadione 2·50, vit. B_12_ 0·02, folic acid 1·17, choline 379, d-pantothenic acid 12·5, riboflavin 7·0, niacin 41·67, thiamine 2·17, d-biotin 0·18, pyridoxine 4·0, ethoxyquin 0·09, Mn 73, Zn 55, Fe 45, Cu 20, I 0·62, Se 0·3.

† Ronozyme Multigrain (xylanase/beta-glucanase; dsm-firmenich).

‡ Ronozyme HiPhos GT 20 000 (dsm-firmenich).

**Table 2.** Protein and carbohydrate analysis of cereals (DM basis)

Item (g/kg)	Maize	Barley	Oats	Wheat
Crude protein	86·2	113	132	138
Acid detergent fibre	39·3	67·6	83·5	40·1
Neutral detergent fibre	121	216	259	152
NSP fractions^*^				
Soluble NSP	7	45	50	20
Insoluble NSP	51	104	169	64
Lignin	1	18	39	10
Starch fractions^*^				
Total starch	651	495	421	577
Rapidly digestible starch	351	182	192	209
Slowly digestible starch	286	301	227	366
Resistant starch	14	11	2	3

* Analysis performed by Englyst Carbohydrates Ltd.

### Data collection and sampling – broilers

Broilers and feed were weighed by pen at the start and end of the experimental period to determine the average daily feed intake and weight gain and feed conversion ratio. On d 24, a 6-h dark period was applied, followed by 2 h of light in the early morning to stimulate feed consumption. Afterwards, all birds were individually weighed, stunned and sacrificed by cervical dislocation.

The birds were then eviscerated, and the gizzards were removed and cut open. Gizzard pH with contents was measured *in situ* by inserting a portable pH metre in the organ. The whole pancreas was excised and weighed. The SI was removed, and the duodenum, jejunum and ileum were excised and separated with the use of clamps to prevent loss of digesta. Duodenum was defined from the site where it emerges from the gizzard to the end of the pancreatic loop; jejunum was defined from the end of the pancreatic loop to 4 cm below Meckel’s diverticulum; ileum was defined as 4 cm below Meckel’s diverticulum and 4 cm above the ileum-cecum-colon junction. A piece of each segment (∼2 cm) from the middle duodenum, jejunum and ileum was cut with scissors and placed in plastic. Subsequently, the entirety of duodenal, jejunal and ileal digesta was collected by gently pushing into plastic containers; a portion of jejunal and ileal digesta (approx. 1·5 g) from each sample was kept in Eppendorf tubes. Contents of birds from the same replicate were pooled. All plastic containers and Eppendorf tubes were immediately snap-frozen with liquid N (–196°C) after sampling. Digesta samples were then kept at −30°C, whereas pancreas, intestinal segments and Eppendorf tubes were stored at −80°C until analysis. The time between the collection of each replicate was 15 min.

### Data collection and sampling – pigs

Pigs were weighed individually at the start and end of the experimental period to calculate the average daily feed intake, average daily weight gain and feed conversion ratio. On day 24, all pigs were fasted for 5 h, followed by 4 h of feed access. All pigs were then weighed before being stunned and sacrificed using Letters Schmidt-Weinberger tongs.

After exsanguination, pigs were then eviscerated, and the stomach, pancreas and SI were excised. A small cut was made in the stomach for insertion of a portable pH metre and measurement of pH *in situ*; the entire content from the stomach was then collected into a plastic container and homogenised, from which a representative sample (approximately 150 g) was collected. A middle segment of the pancreas was excised and placed in a plastic bag. The SI was divided into duodenum, jejunum and ileum following the description of König and Liebich^(29)^: duodenum was defined from the pylorus to the end of the region held by the duodenocolic fold (approximately 60 cm from the pylorus); ileum was defined from the beginning of the region held by the ileocaecal fold to approximately 5 cm before the ileocaecal junction; the remaining segment was considered jejunum. Prior to the collection of digesta, a 2 cm segment was cut from the middle section of each organ, and contents were removed and stored in plastic bags. The entire digestive content of each segment was then collected by gently pushing it into plastic containers. Due to the large amount of digesta in the jejunum, it was first gathered into a larger container and homogenised before collecting representative samples (approx. 150 g). A portion (approx. 1·5 g) of jejunal and ileal digesta was held in Eppendorf tubes. All containers were immediately snap-frozen in liquid N (–196°C) after sampling. Afterwards, plastic containers with digesta samples were kept at –30°C, and pancreas, intestinal segments and Eppendorf tubes with digesta were kept at –80°C until analysis. The time between the collection of each replicate was 15 min, akin to the broiler sampling procedure.

### Chemical analyses

Cereal and feed samples were ground to pass through a sieve with a mesh size of 0·5 mm (Retsch, Ultra Centrifugal Mill ZM 200) and analysed in duplicate for DM (overnight oven-drying at 105°C), CP (method 976·05), aether extract (method 920·39), acid detergent fiber (method 942·05, expressed inclusive of residual ash) and neutral detergent fiber (method 973·18, assayed with heat-stable amylase and expressed inclusive of residual ash) according to the Association of Official Agricultural Chemists (2005). Soluble and insoluble NSP in cereals and diets were determined according to Englyst *et al.* ^(30)^. Dietary nitrogen content was analysed using a KjelFoss Automatic 16 210 analyser (A/S N. Foss Electric), and aether extract was determined using a Soxtec System HT 1043 Extraction Unit (Foss Tecator). Gross energy was determined using an adiabatic bomb calorimeter (KL 12Mn, Precyzja-Bit PPHU) standardised with benzoic acid. Starch content was determined utilising thermostable *α*-amylase and amyloglucosidase commercial kits (Megazyme International) according to the Association of Official Agricultural Chemists (method 996·11). Starch fractions (rapidly digestible starch, slowly digestible starch, available starch, resistant starch) in cereals and diets were determined using the method of Englyst *et al.* ^(31)^. Content of TiO_2_ in the diets was determined according to Short *et al.* ^(32)^.

Prior to analysis, all digesta samples were freeze-dried (Christ Epsilon-10D LSC plus, Medizinischer Apparatebau). Jejunal and ileal digesta were then analysed for DM, CP, total starch and TiO_2_ using the previously described methods. Additionally, total starch in the jejunum was also analysed using the variation of the Association of Official Agricultural Chemists (method 996·11) for samples containing d-glucose and/or maltodextrins, by rinsing the samples twice with 10 ml of aqueous ethanol (80 % v/v), centrifuging for 10 min at 1800 ***g*** between each rinsing and discarding the supernatant. This was done to investigate possible differences between broilers and pigs regarding the presence of non-absorbed free glucose at the jejunal level.

### Particle size distribution of digesta

After freeze-drying, all duodenal contents and approximately 5 g of jejunal contents from both pigs and broilers were used to determine PSD by a laser diffraction method on a Malvern Mastersizer S instrument (Malvern Instruments Ltd), detecting particle diameters in the range from 0·05 to 2000 µm. All samples were remoisturized in a beaker with deionised water for 5 min before entering the instrument. The instrument provided PSD information expressed as calculated volume percentages of particles less than 2000 µm in size.

### Ileal viscosity and amylase and lipase activities

After collection of ileal digesta from broilers and pigs, approximately 2 g (wet weight) from each sample were immediately centrifuged at 12 700 ***g*** for 5 min. The supernatant was withdrawn and viscosity (mPas·s = cP = 1 × 100 dyne s cm^–2^) was determined using a Brookfield Digital DV-II+ cone/plate viscometer (Brookfield Engineering Laboratories Inc.) at a shear rate of 42·5 s^–1^ at 40°C.

Approximately 100 µg of frozen pancreases and jejunal and ileal chymes were weighed, mixed with PBS and homogenised, and the homogenates were centrifuged at 10 000 ***g*** for 30 min at 4°C. For analysis of amylase, supernatants were diluted fifty times; for lipase, pancreas supernatants were diluted fifty times, whereas jejunal and ileal supernatants were not diluted. Amylase and lipase activity measurements were carried out using colorimetric assay kits (BioVision). The results were quantified in terms of glycerol (for lipase) and nitrophenol (for amylase) released and expressed per g of pancreas and per g of DM of jejunal and ileal digesta.

### Real-time quantitative PCR

RNA was extracted from homogenised pancreas and jejunal tissue using Extrazol (DNA Gdansk) according to the manufacturer’s instructions and reverse transcribed into cDNA with a high-capacity cDNA reverse transcription kit (Life Technologies). The mRNA expression levels of amylase and lipase in the pancreas and SGLT1, GLUT2 and GLP-1 in the jejunum were then measured by real-time qPCR using HOT FIREPol EvaGreen (Solis Biodyne) as a DNA binding dye and performed in a Quant Studio 12K Flex™system. In this study, *β*-actin gene was selected as a reference gene due to its stable expression across samples. The primers (Table 3) were designed in Primer-BLAST (National Institute of Health). The relative expression levels were normalised to the *β*-actin gene and expressed as relative expression of target gene per reference gene and calculated using the 2−Δ Ct method^(33)^.

**Table 3.** Sequence of genes used in RT-PCR

Species	Gene	Primer sequence (F: forward primer; R: reverse primer)	Product size (bp)
Chicken	B-actin	F: CACAGATCATGTTTGAGACCTT R: CATCACAATACCAGTGGTACG	101
	Lipase	F: TCATACTCTTCAGCCAATGTCC R: GGGTCCAGTCCAGTTATTCTTC	110
	Amylase	F: TCACAGGCAGTCAGTACTTTG R: GTAGGCCATCTTCTCTCCATTC	104
	SGLT-1	F: GTCCTGGCAGTGGGAGTATG R: AAGAGTGAAGCACCGATCGG	108
	GLUT2	F: CACACTATGGGCGCATGCT R: ATTGTCCCTGGAGGTGTTGGTG	68
	GLP-1	F: CCAAGCGTCATTCTGAATTTG R: TGACCTTCCAAATAAGAGGTGATA	76
Pig	B-actin	F: CGAGGCCCAGAGCAAGAG R: TCCATGTCGTCCCAGTTGGT	81
	Lipase	F: GGCTCCCGAACTGGATACAC R: GATCCAGCCCTGTGATTCGT	205
	Amylase	F: CTGCTGCTTTCAGCCTTTGG R: ACCGCTCACATTCAAGAGCA	124
	SGLT1	F: CCACTTTCCCTATAAAACCTCAC R: CTCCATCAAACTTCCATCCTCAG	151
	GLUT2	F: CCTGCTTGGTCTATCTGCTGTG R: TTGATGCTTCTTCCCTTTCTTT	194
	GLP1	F: CTGCACAAGGACAACTCCAG R: GCTTGGATTCCTCACACTCG	61

### Calculations and statistical analysis

The following equation was used to calculate coefficients of jejunal and ileal apparent nutrient digestibility:

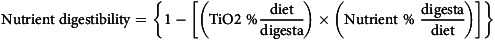




One cage (two broilers) or one pig was considered the experimental unit. The residue normality of the data was determined by the Shapiro–Wilk test. The effect of diets on growth performance variables of broilers and pigs was analysed apart as a one-way ANOVA, and all other variables were submitted to a two-way ANOVA to study the effect of diet, animal species and their interaction assuming significance at *P* < 0·05 and tendency at 0·05 < *P* ≤ 0·1. When significant, the effect of diet and interactions were submitted to the Tukey test for mean comparison. All statistical procedures were conducted on SAS statistical software (version 9.4, SAS Institute Inc.). The sample size was validated through a retrospective power analysis (G * Power 3·1, Heinrich Heine University Düsseldorf) using the variation in ileal starch digestibility, considered one of the primary outcomes of the study. A statistical power of 0·67 and 0·78 were achieved for the main effects of species and cereals, respectively, whereas the species × cereal interaction had a power of 0·74.

## Results

### Growth performance

For broilers, average daily weight gain was not affected (Table 4), but birds fed oat-based diets had a higher average daily feed intake (*P* < 0·05) than those fed the barley-based diet, which then resulted in the highest feed conversion ratio (*P* < 0·05) compared with birds fed maize and barley diets. For pigs, growth performance was not affected by dietary treatments. Both species presented normal growth across all diets according to breeders’ performance guidelines^(34,35)^.

**Table 4.** Growth performance of broilers and pigs fed diets based on different cereals

Diet	Average daily feed intake (g)	Average daily weight gain (g)	Feed conversion ratio (g/g)
Broiler chickens (14–24 d old)			
Maize	106^ab^	78·7	1·342^b^
Barley	103^b^	75·5	1·367^b^
Oat	110^a^	75·1	1·467^a^
Pooled sem	1·47	1·16	0·041
*P*	0·005	0·061	< 0·001
Pigs (50–60 d old)			
Maize	1·204	672	1·842
Barley	1·231	634	1·987
Oat	1·246	702	1·785
Pooled sem	28·5	34·8	0·260
*P*	0·584	0·398	0·221

^a,b^Values within a row with different superscripts differ significantly at *P* < 0·05.

### Nutrient digestibility, pH of gizzard and stomach and ileal viscosity

In the jejunum, pigs fed barley exhibited lower DM digestibility compared with the other cereals (*P* < 0·05; Table 5), while for broilers, the maize diet had a higher DM digestibility than the other cereals (*P* < 0·05), resulting in an interaction (*P* < 0·05). A similar interaction was noted for CP digestibility (*P* < 0·05). In the ileum, no interaction was detected for DM and CP digestibility. Maize-based diets resulted in the highest ileal DM and CP digestibility, followed by barley, and then oats. Broilers had greater ileal DM and CP digestibility compared with pigs (*P* < 0·05).

**Table 5.** Apparent digestibility of DM, crude protein and starch, pH of gizzard and stomach and ileal viscosity of broilers and pigs fed diets based on different cereals

Species	Diet	DM content (%)		Apparent DM digestibility		Apparent CP digestibility		Apparent starch digestibility			Gizzard/stomach pH	Ileal viscosity (cP)
		Jejunum	Ileum	Jejunum	Ileum	Jejunum	Ileum	Jejunum	Jejunum (ethanol-rinsed)^*^	Ileum		
Broiler chicken	Maize	16·4^b^	18·3	0·63^a^	0·75	0·57^a^	0·79	0·87	0·91	0·96^ab^	3·73	2·47^b^
	Barley	15·9^b^	18·4	0·53^b^	0·69	0·50^ab^	0·76	0·81	0·83	0·94^b^	3·59	2·73^ab^
	Oat	18·1^a^	18·9	0·49^b^	0·64	0·41^bc^	0·71	0·90	0·95	0·99^a^	3·72	3·11^a^
Pig	Maize	9·86^c^	9·40	0·56^ab^	0·75	0·49^ab^	0·73	0·89	0·91	0·97^a^	4·60	1·35^c^
	Barley	9·39^c^	9·01	0·41^c^	0·68	0·34^c^	0·67	0·81	0·84	0·96^ab^	4·52	1·54^c^
	Oat	7·56^c^	7·48	0·51^b^	0·56	0·45^bc^	0·61	0·93	0·96	0·98^a^	4·32	1·37^c^
Pooled sem		0·608	0·709	0·022	0·013	0·023	0·012	0·014	0·003	0·003	0·145	0·091
Effect of species												
Broiler chicken		16·8	18·5	0·55	0·69	0·49	0·75	0·86	0·89	0·96	3·68	2·77
Pig		8·93	8·63	0·50	0·66	0·43	0·67	0·88	0·90	0·97	4·48	1·42
Effect of diet												
	Maize	13·1	13·8	0·59	0·75^a^	0·53	0·76^a^	0·88^b^	0·91^b^	0·97	4·17	1·91
	Barley	12·6	13·7	0·47	0·69^b^	0·42	0·71^b^	0·81^c^	0·84^c^	0·95	4·05	2·14
	Oat	12·8	13·2	0·50	0·60^c^	0·43	0·66^c^	0·92^a^	0·96^a^	0·98	4·02	2·24
*P*												
Species		< 0·001	< 0·001	0·001	0·040	0·004	< 0·001	0·205	0·391	0·086	< 0·001	< 0·001
Diet		0·845	0·650	< 0·001	< 0·001	0·001	< 0·001	< 0·001	< 0·001	0·003	0·593	0·004
Interaction		0·024	0·170	0·001	0·165	0·0015	0·584	0·581	0·635	0·024	0·487	0·003

CP, crude protein; sem, standard error of the mean.

^a,b^Values within a row with different superscripts differ significantly at *P* < 0·05.

* Analysed by a variation of the method 996·11 by the Association of Official Agricultural Chemists for samples containing d-glucose and/or maltodextrins.

Regarding starch digestibility at the jejunal level, no interactions or species-based differences were observed. Oat-based diets led to higher jejunal starch digestibility, followed by maize and barley (*P* < 0·05). When the samples were rinsed with ethanol, the coefficients for jejunal starch digestibility were higher, but the statistical outcome remained the same. In the ileum, an interaction was observed, showing that barley resulted in lower starch digestibility compared with oats only for broilers (*P* < 0·05).

Pigs had a higher pH in the stomach area than broilers (*P* < 0·05), but dietary treatments did not affect the pH. Ileal viscosity was consistently lower in pigs than in broilers (*P* < 0·05), but diet only affected viscosity in broilers, where oats gave higher viscosity (*P* < 0·05) than maize, thus resulting in an interaction (*P* < 0·05).

### Particle size distribution of digesta

The duodenal digesta in broilers featured a higher concentration of particles < 0·1 mm compared with pigs (*P* < 0·05; Table 6). In contrast, pigs had a higher (*P* < 0·05) percentage of particles within the range of 0·2–2 mm. A dietary influence was also observed, showing that maize-based diets resulted in a larger proportion of particles below 0·1 mm compared with oat-based diets. Conversely, oat diets led to an increased presence of particles ranging from 0·2 to 0·5 mm compared with maize and barley diets.

**Table 6.** Particle size distribution of duodenal digesta of broilers and pigs fed diets based on different cereals, expressed as calculated volume percentage

Species	Diet	Volume of particles (%)					
		0–0·1 mm	0·1–0·2 mm	0·2–0·5 mm	0·5–1 mm	1–1·6 mm	1·6–2 mm
Broiler chicken	Maize	73·9	13·2	10·8	1·75	0·23	0·01
	Barley	71·9	13·9	11·8	2·05	0·29	0·01
	Oat	68·2	14·9	13·4	3·14	0·28	0·01
Pig	Maize	43·2	14·4	26·2	13·6	2·53	0·11
	Barley	39·9	12·9	25·7	17·1	4·22	0·20
	Oat	31·2	15·9	32·8	16·6	3·38	0·15
Pooled sem		2·812	0·753	1·511	1·570	0·603	0·033
Effect of species							
Broiler chicken		71·40	14·00	12·01	2·31	0·27	0·01
Pig		38·10	14·39	28·22	15·76	3·38	0·15
Effect of diet							
	Maize	58·6^a^	13·8^ab^	18·5^b^	7·68	1·38	0·06
	Barley	55·9^ab^	13·4^b^	18·7^b^	9·57	2·26	0·11
	Oat	49·7^b^	15·4^a^	23·1^a^	9·86	1·83	0·08
*P*							
Species		< 0·001	0·592	< 0·001	< 0·001	< 0·001	< 0·001
Diet		0·008	0·023	0·005	0·328	0·352	0·306
Interaction		0·513	0·312	0·186	0·604	0·407	0·313

sem, standard error of the mean.

^a,b^Values within a row with different superscripts differ significantly at *P* < 0·05.

In the jejunum (Table 7), the distinction of PSD between species was not detected. There was a lower (*P* < 0·05) proportion of particles below 0·1 mm when barley was fed than for the other cereals and an increased proportion of intermediate-sized particles (0·2–0·5 mm) compared with maize (*P* < 0·05). Other PSD categories exhibited interactions where oats diets gave a greater (*P* < 0·05) volume of smaller particles (0·1–0·2 mm) than the other cereals for broilers only. A similar interaction was observed for particles between 0·5 and 1·6 mm, where oats had a smaller proportion than other cereals for broilers only.

**Table 7.** Particle size distribution of jejunal digesta of broilers and pigs fed diets based on different cereals, expressed as calculated volume percentage

Species	Diet	Volume of particles (%)					
		0–0·1 mm	0·1–0·2 mm	0·2–0·5 mm	0·5–1 mm	1–1·6 mm	1·6–2 mm
Broiler chicken	Maize	30·4	16·1^b^	29·2	18·9^a^	4·97^a^	0·31
	Barley	26·5	16·3^b^	32·1	20·1^a^	4·85^a^	0·22
	Oat	32·3	19·9^a^	31·8	13·3^b^	2·48^b^	0·10
Pig	Maize	33·7	16·6^b^	28·9	16·8^ab^	3·92^ab^	0·17
	Barley	24·6	16·2^b^	32·7	20·6^a^	5·44^a^	0·36
	Oat	30·9	17·4^ab^	28·5	18·0^ab^	4·87^a^	0·22
Pooled sem		2·112	0·621	1·147	1·278	0·552	0·072
Effect of species							
Broiler chicken		29·8	17·4	31·0	17·4	4·10	0·21
Pig		29·7	16·7	30·1	18·5	4·74	0·25
Effect of diet							
	Maize	32·0^a^	16·3	29·1^b^	17·8	4·45	0·24
	Barley	25·5^b^	16·2	32·4^a^	20·4	5·15	0·29
	Oat	31·7^a^	18·6	30·2^ab^	15·7	3·68	0·16
*P*							
Species		0·999	0·172	0·289	0·336	0·161	0·477
Diet		0·004	0·003	0·016	0·002	0·036	0·158
Interaction		0·410	0·041	0·206	0·032	0·011	0·075

sem, standard error of the mean.

^a,b^Values within a row with different superscripts differ significantly at *P* < 0·05.

### Amylase and lipase activity in the pancreas and digesta

The activity of pancreatic amylase (Table 8) was only influenced by diets (*P* < 0·05), where reduced amylase activity was observed when fed oats compared with barley-based diets. In the jejunal digesta, oat-based diets also led to lower amylase activity per g of DM content compared with barley-based diets, although the effect tended (*P* = 0·064) to mainly be present in broilers. In the ileum, pigs exhibited greater amylase activity than broilers (*P* < 0·001), again with a tendency for a reduction in amylase activity with oats compared with the other cereals for broilers only (*P* = 0·088).

**Table 8.** Amylase and lipase activity in the pancreas and in jejunal and ileal digesta from broilers and pigs fed diets based on different cereals

Species	Diet	Pancreas (U/g of pancreas)		Jejunum (U/g DM)		Ileum (U/g DM)	
		Amylase	Lipase	Amylase	Lipase	Amylase	Lipase
Broiler chicken	Maize	155	7·43	323	6·45^b^	80·1	2·81^c^
	Barley	180	6·11	357	6·00^b^	94·9	1·68^c^
	Oat	152	4·24	198	5·76^b^	40·7	1·02^c^
Pig	Maize	181	25·9	268	7·35^b^	187	5·96^b^
	Barley	230	28·3	300	6·87^b^	184	7·44^ab^
	Oat	137	26·8	284	9·09^a^	219	9·41^a^
Pooled sem		19·75	1·578	33·04	0·39	20·88	0·55
Effect of species							
Broiler chicken		162	5·93	293	6·07	71·9	1·84
Pig		183	27·0	284	7·77	196	7·60
Effect of diet							
	Maize	168^ab^	16·6	296^ab^	6·90	133	4·38
	Barley	205^a^	17·2	328^a^	6·43	139	4·56
	Oat	144^b^	15·5	241^b^	7·43	129	5·22
*P*							
Species		0·213	< 0·001	0·757	< 0·001	< 0·001	< 0·001
Diet		0·001	0·553	0·036	0·046	0·898	0·290
Interaction		0·225	0·255	0·064	< 0·001	0·086	0·001

sem, standard error of the mean.

^a,b^Values within a row with different superscripts differ significantly at *P* < 0·05.

Lipase activity in the pancreas was consistently higher in pigs than in broilers, regardless of dietary treatments (*P* < 0·001). In the jejunum, an interaction showed that pigs fed oat-based diets exhibited higher lipase activity compared with other cereals, while no such effect was observed for broilers (*P* < 0·05). In the ileum, elevated lipase activity was observed in pigs in relation to broilers, with an interaction between species and cereals type due to an elevated level for oat diets only seen in pigs (*P* < 0·05).

### Expression of pancreatic enzymes and GLUT

The relative mRNA expression of amylase and lipase in the pancreas and of SGLT-1, GLUT2 and GLP-1 in the jejunum was not affected by any interactions (Table 9). Expression of pancreatic amylase was similar between species, whereas expression of pancreatic lipase tended (*P* = 0·08) to be higher in pigs. SGLT-1 mRNA levels were higher in the jejunum of pigs than in broilers (*P* < 0·001); in contrast, broilers showed higher levels of GLUT2 mRNA in the jejunum (*P* < 0·001). Relative expression of GLP-1 in the jejunum tended (*P* = 0·07) to be higher in pigs. The different cereals only had an influence on the relative expression of pancreatic amylase mRNA, which was higher when barley diets were fed compared with maize (*P* < 0·05).

**Table 9.** Relative mRNA expression of pancreatic enzymes, GLUT and glucagon-like peptide-1 hormone in the jejunum of broiler chickens and pigs fed diets based on different cereals

Species	Diet	Pancreas^*^		Jejunum^*^		
		Amylase	Lipase	SGLT-1	GLUT2	GLP-1
Broiler chicken	Maize	0·48	6·68	1·41	9·99	3·44
	Barley	0·53	6·11	1·46	7·83	5·33
	Oat	0·52	4·09	1·17	11·6	6·20
Pig	Maize	0·53	8·06	11·6	0·02	5·77
	Barley	0·55	8·73	10·4	0·01	7·11
	Oat	0·52	7·73	17·2	2·23	8·47
Pooled sem		0·004	0·750	1·305	1·042	0·595
Effect of species						
Broiler chicken		0·50	5·63	1·35	9·80	4·99
Pig		0·53	8·17	13·0	0·76	7·11
Effect of diet						
	Maize	0·51^b^	7·37	6·51	5·01	4·60
	Barley	0·54^a^	7·42	5·90	3·92	6·22
	Oat	0·52^ab^	5·91	9·17	6·91	7·34
*P*						
Species		0·022	0·082	< 0·001	< 0·001	0·076
Diet		0·017	0·620	0·256	0·250	0·170
Interaction		0·246	0·809	0·202	0·823	0·978

sem, standard error of the mean.

^a,b^Values within a row with different superscripts differ significantly at *P* < 0·05.

* mRNA relative expression normalised with *β*-actin value and expressed as relative expression of target gene/*β*-actin per 1 µg of RNA, calculated through 2−Δ Ct method.

## Discussion

To find a common basis for comparing digestive physiology and nutrient digestibility between broilers and pigs is a challenging task considering their particularities regarding optimal feed particle size, nutritional requirements and anatomical differences of the gastrointestinal tract. In this study, we aimed at a grinding size of diets between 500 and 600 µm, based on recommendations for growing pigs and its effects on performance and nutrient utilisation^(36)^. This represented a compromise, since the particle size recommendation for poultry is higher, for example, around 900 µm^(37)^, although broilers also seem to perform well with finer grinding^(38)^. Although the diets were not identical in terms of nutritional levels due to inherent variations in the cereals, we were able to standardise the starch proportions of each main cereal by adjusting their inclusion level and incorporating wheat as a secondary carbohydrate source in all three diets. This approach was necessary to isolate the effects of the different cereals and enable the investigation of cereal–species interaction focused on starch digestibility.

The distinction between SI segments is another item of disparity between both species. In poultry, the anatomical division of duodenum, jejunum and ileum is clearer, with the duodenal loop and Meckel’s diverticulum generally used as landmarks^(39)^. In pigs, morphological features of the three SI segments may be less distinct^(40)^, hence why many studies choose to simply partition the SI into equal parts^(41)^ or to employ cannulation techniques^(42)^ without describing segments. Our study followed the description of König and Liebich^(29)^ for the separation of SI regions, matching the proportions indicated by Laerke and Hedemann^(40)^: the duodenum and ileum each representing 4–5 % and jejunum around 90 % of the pigs’ SI. Comparatively, in 21-d-old broiler chickens, the duodenum may account for approximately 15–16 % and jejunum and ileum approximately 40–42 % of the SI^(43)^.

The main hypothesis for the conceptualisation of this study was that broiler chickens would exhibit a superior starch digestibility to growing pigs, based on the consistently high coefficients observed in broiler studies^(10)^. Then, by comparing them with pigs, both monogastric species accustomed to starch as their primary energy source, but with distinct digestive systems and digestion tactics, we aimed to explore some of the mechanisms underlying the high starch digestion capacity of poultry. This hypothesis was rejected, as starch digestibility across the jejunal and ileal sites was similar between both species. Notwithstanding this, different aspects related to the digestive process between species were identified and are discussed herein, along with the subtle species–cereals interactions observed in some variables.

Our first remarks concern the distinct function of the anterior tract during digestion in broilers and pigs. Broilers had a lower gizzard pH compared with the stomachs of pigs. As denoted by Lee *et al.* ^(44)^, pH in the gizzard fluctuates between 0·6 and 3·8, whereas pH in the stomach of weaned pigs can be slightly higher, varying from 2·6 to 5·0 due to the transition from liquid milk to highly buffering solid diets and an underdeveloped HCl secretion^(45)^. A low gastric pH may influence starch digestibility due to the breakdown of proteins surrounding the starch granules, mainly prolamins^(46)^. Broilers also had a greater proportion of particles smaller than 0·1 mm in the duodenum compared with pigs (71 × 38 %), indicating the gizzard’s action on further reducing particle size of the digesta before entry into the SI. The avian gizzard is reported to consistently grind feed particles down to sizes as small as < 40 µm, regardless of the original feed structure^(22,37,47)^. In contrast, pigs rely on mastication, whose grinding capability can be limited up to around 4 months of age^(48)^, along with a gentler gastric motility and grinding function compared with other mammals^(40,48)^. Compared with poultry, data on PSD of digesta throughout the gastrointestinal tract of pigs are sparse. Gao *et al.* ^(49)^ observed a proportion of 55 % of particles < 0·072 mm in the duodenum of cannulated barrows fed maize with 682 µm mean particle size, a slightly higher proportion of small particles compared with our observations. Moving towards the jejunum, PSD differences between species were less evident, suggesting that the high proportion of small particles in the duodenum of broilers have been rapidly digested at this point due to their increased surface area^(37)^.

Even though starch digestion was similar between species, broilers showed higher ileal CP digestibility. Comparative studies by Park *et al.* ^(50,51)^ reported higher standardised ileal CP digestibility in pigs relative to broilers, attributed to a slower passage rate of feed through the SI. Adedokun and Adeola^(52)^ observed that ileal endogenous AA losses were similar between broilers and pigs but influenced more by dietary factors, that is, different N sources and fibre content. Despite a longer retention time of feed in the gastrointestinal tract of pigs than in poultry^(18,19)^, other factors such as reflux of digesta and gizzard grinding combined with a more acidic pH may have contributed to an increased CP digestion, thereby increasing the digestibility of DM as well. However, the dynamics of protease activity between species warrant further investigation.

In the ileum, pigs had a less viscous digesta than broilers, and unlike broilers, ileal viscosity of pigs was not affected by the cereals. As argued by Moran^(21)^, a more viscous intestinal digesta in poultry results from a higher DM content, making their digestive function more sensitive to changes in diet viscosity. Notably, the ileal starch digestibility of barley was lower only in broilers, likely due to its viscous fibre content^(6)^. Even though oats produced a similarly viscous ileal digesta in broilers, oat starch digestibility was not impaired the same way presumably due to its lower content of resistant starch. Our findings agree with Takahashi and Sakata^(53)^ and Takahashi *et al.* ^(54)^, who found chicken digesta to be more viscous than in pigs, although in the caeca. Lentle *et al.* ^(55)^ suggest that low viscosity in the tract of pigs may prompt a better mixing and dilution of digesta with pancreatic secretions. Notably, all diets were supplemented with a blend of xylanase and glucanase to counter the high fibre contents from barley and oats, as these enzymes are able to reduce digesta viscosity in broilers^(56)^ and pigs^(57)^. Bedford and Schulze^(58)^ noted that greater intestinal viscosity increases the response to fibre-degrading enzymes. While we can only speculate whether a viscosity-reducing enzyme effect was more relevant to broilers than pigs due to the lower moisture content of the digesta, a direct comparison between species could identify possible differences in enzymatic efficiency, as none appear to have been reported. Wheat effects on ileal viscosity were not a concern due to its relatively low soluble NSP content and moderate inclusion.

Studies that measured enzyme activity in U/ml of intestinal contents suggest that amylase activity is higher in broilers, for example, 268 in the duodenum + jejunum^(59)^ *v*. 162 in the duodenum and 25 U/ml in the ileum of growing pigs^(60)^ without exogenous enzyme supplementation. However, different studies are susceptible to variability in sample handling and storage conditions^(61)^. To account for differences in DM of digesta between broilers and pigs, we expressed enzyme activity as U per g of DM content. Amylase had similar activity in the jejunum of both species, where amylolytic action reaches its peak^(48,62)^, but was higher in the ileum of pigs. This implies that amylase activity in the ileum of broilers was more quickly reduced following a rapid starch digestion, which may also relate to the lower amylase mRNA expression in the broiler pancreas through feedback regulation. Some of the activity in the ileum of pigs may represent salivary amylase, which is absent in broilers but plays a relevant role in starch digestion in the upper gut of pigs and can remain active in a higher pH range^(63)^. In both the ileum and pancreas, lipase activity was higher for pigs, along with a tendency for higher mRNA expression of pancreatic lipase. This denotes a high lipolytic capacity of post-weaned pigs described by Liu *et al.* ^(64)^, due to their adaptation for digesting fat-rich (7–10 %) sow milk^(65)^. Conversely, low pancreatic lipase activity and limited bile secretion have been described in young birds^(66,67)^. A high lipase activity can influence starch digestion through the breakdown of lipid–amylose complexes coating the starch granules^(68)^. Furthermore, the higher ileal lipase activity in barley and oat-fed pigs reflects the higher addition of oil in these diets, which required more lipolysis.

Our investigation of relative mRNA expression of SGLT-1 and GLUT2, the main GLUT in the SI of birds and mammals, has shown an interesting relation where SGLT-1 expression in the jejunum was higher in pigs, while GLUT2 predominated in broilers. For an in-depth review of the properties of SGLT-1 and GLUT2, we recommend the reviews by Sano *et al.* ^(69)^ and Röder *et al.* ^(70)^. In brief, GLUT2 mediates passive transmembrane transport of glucose, corroborating the remarks of Mcwhorter *et al.* ^(20)^ that suggested an extensive paracellular nutrient absorption in birds exceeding that in mammals. Also, because uptake of glucose through GLUT2 occurs when luminal levels are high, this could be tied to a rapid starch digestion in birds, leading to a swift release of glucose that upregulates GLUT2 expression. However, we found no differences between free glucose concentration in jejunal digesta of both species when comparing starch digestibility in ethanol-rinsed *v*. non-rinsed samples. In contrast, Byers *et al.* ^(71)^ reported similar SGLT-1 and GLUT2 expression in both birds and mammals. Moreover, relative mRNA expression was assessed in relation to different reference genes for each species, which may limit their comparability.

In conclusion, broilers and pigs were similarly efficient at digesting starch, showing that starch utilisation capacity is more related to its dietary source. Differences between species indicated that nutrient digestion efficiency in broilers could be attributed to a lower gastric pH and further reduction of feed particle size by gizzard grinding, while pigs were characterised by having a less viscous digesta and higher lipase activity. Future comparative studies could help elucidate differences in feed retention capability and gastrointestinal transit time to further extend our comprehension of digestion kinetics between these two species.

## References

[1] Moran ET (2019) Starch: granule, amylose-amylopectin, feed preparation, and recovery by the fowl’s gastrointestinal tract. J Appl Poult Res 28, 566–586.

[2] Magallanes-Cruz PA , Flores-Silva PC & Bello-Perez LA (2017) Starch structure influences its digestibility: a review. J Food Sci 82, 2016–2023. 28753728 10.1111/1750-3841.13809

[3] Zaefarian F , Abdollahi MR & Ravindran V (2015) Starch digestion in broiler chickens fed cereal diets. Anim Feed Sci Tech 206, 16–29.

[4] Svihus B , Uhlen AK & Harstad OM (2005) Effect of starch granule structure, associated components and processing on nutritive value of cereal starch: a review. Anim Feed Sci Tech 122, 303–320.

[5] Kaczmarek SA , Cowieson AJ , Józefiak D , et al. (2014) Effect of maize endosperm hardness, drying temperature and microbial enzyme supplementation on the performance of broiler chickens. Anim Prod Sci 54, 956–965.

[6] Jacob JP & Pescatore AJ (2012) Using barley in poultry diets-a review. J Appl Poult Res 21, 915–940.

[7] Zwer P (2010) Oats: characteristics and quality requirements. In Cereal Grains, 1st ed., pp. 163–182 [ CW Wrigley and IL Batey , editors]. Sawston, UK: Woodhead Publishing.

[8] Svihus B & Hetland H (2001) Ileal starch digestibility in growing broiler chickens fed on a wheat-based diet is improved by mash feeding, dilution with cellulose or whole wheat inclusion. Br Poult Sci 42, 633–637. 11811915 10.1080/00071660120088461

[9] Zelenka J & Čerešňáková Z (2005) Effect of age on digestibility of starch in chickens with different growth rate. Czech J Anim Sci 50, 411–415.

[10] Svihus B (2014) Starch digestion capacity of poultry. Poult Sci 93, 2394–2399. 25012853 10.3382/ps.2014-03905

[11] Bassi LS , Hejdysz M , Pruszyńska-Oszmalek E , et al. (2023) The effect of amylase supplementation on individual variation, growth performance, and starch digestibility in broiler chickens. Poult Sci 102, 102563. 36871332 10.1016/j.psj.2023.102563 PMC9995474

[12] Zhang YC , Jørgensen H , Fernandez JA , et al. (2004) Digestibility of carbohydrates in growing pigs: a comparison between the T-cannula and the steered ileo-caecal valve cannula. Arch Anim Nutr 58, 219–231. 15264671 10.1080/00039420410001701396

[13] Yin F , Zhang Z , Huang J , et al. (2010) Digestion rate of dietary starch affects systemic circulation of amino acids in weaned pigs. Br J Nutr 103, 1404–1412. 20102672 10.1017/S0007114509993321

[14] Amaral NO , Amaral LGM , Cantarelli VS , et al. (2015) Influence of maize particle size on the kinetics of starch digestion in the small intestine of growing pigs. Anim Prod Sci 55, 1250–1254.

[15] Selle PH , Moss AF , Khoddami A , et al. (2021) Starch digestion rates in multiple samples of commonly used feed grains in diets for broiler chickens. Anim Nutr 7, 450–459. 34258433 10.1016/j.aninu.2020.12.006 PMC8245903

[16] Cervantes-Pahm SK , Liu Y & Stein HH (2014) Comparative digestibility of energy and nutrients and fermentability of dietary fibre in eight cereal grains fed to pigs. J Sci Food Agric 94, 841–849. 23893839 10.1002/jsfa.6316

[17] Wiseman J (2006) Variations in starch digestibility in non-ruminants. Anim Feed Sci Tech 130, 66–77.

[18] Svihus B & Itani K (2019) Intestinal passage and its relations to digestive processes. J Appl Poult Res 28, 546–555.

[19] Wilfart A , Montagne L , Simmins H , et al. (2007) Effect of fibre content in the diet on the mean retention time in different segments of the digestive tract in growing pigs. Livest Sci 109, 27–29.

[20] Mcwhorter TJ , Caviedes-Vidal E & Karasov WH (2009) The integration of digestion and osmoregulation in the avian gut. Biol Rev 84, 533–565. 19673857 10.1111/j.1469-185X.2009.00086.x

[21] Moran ET (2022) *Poultry and swine gastrointestinal systems functionally differ to influence feedstuff digestion and responses to supplemental enzymes* . In Enzymes in Farm Animal Nutrition, 3rd ed., pp. 220–239 [ MR Bedford , et al., editors]. Wallingford: CAB International.

[22] Svihus B (2011) The gizzard: function, influence of diet structure and effects on nutrient availability. Worlds Poult Sci J 67, 207–223.

[23] Sacranie A , Svihus B , Denstadli V , et al. (2012) The effect of insoluble fibre and intermittent feeding on gizzard development, gut motility, and performance of broiler chickens. Poult Sci 91, 693–700. 22334745 10.3382/ps.2011-01790

[24] Rodgers NJ , Choct M , Hetland H , et al. (2012) Extent and method of grinding of sorghum prior to inclusion in complete pelleted broiler chicken diets affects broiler gut development and performance. Anim Feed Sci Tech 171, 60–67.

[25] Rojas OJ & Stein HH (2017) Processing of ingredients and diets and effects on nutritional value for pigs. J Anim Sci Biotech 8, 48. 10.1186/s40104-017-0177-1 PMC5452379 28572976

[26] Solà-Oriol D , Roura E & Torrallardona D (2009) Feed preference in pigs: relationship with feed particle size and texture. J Anim Sci 87, 571–582. 18952742 10.2527/jas.2008-0951

[27] Aviagen (2022) Ross 308 Broiler: Nutrition Specifications. Newbridge, Midlothian, Scotland, UK: Aviagen Limited.

[28] National Research Council (2012) Nutrient Requirements of Swine. Washington, DC: The National Academic Press.

[29] König HE & Liebich H (editors) (2020) Veterinary Anatomy of Domestic Animals, 7th ed. Stuttgart, Germany: Schattauer GmbH.

[30] Englyst HN , Quigley ME & Hudson GJ (1994) Determination of dietary fibre as non-starch polysaccharides with gas-liquid chromatographic, high-performance liquid chromatographic or spectrophotometric measurement of constituent sugars. Analyst 119, 1497–1509. 7943740 10.1039/an9941901497

[31] Englyst KN , Englyst HN , Hudson GJ , et al. (1999) Rapidly available glucose in foods: an *i*n vitro measurement that reflects the glycemic response. Am J Clin Nutr 69, 448–454. 10075329 10.1093/ajcn/69.3.448

[32] Short FJ , Gorton P , Wiseman J , et al. (1996) Determination of titanium dioxide added as an inert marker in chicken digestibility studies. Anim Feed Sci Tech 59, 215–221.

[33] Livak KJ & Schmittgen TD (2001) Analysis of relative gene expression data using real-time quantitative PCR and the 2-ΔΔCT method. Methods 25, 402–408. 11846609 10.1006/meth.2001.1262

[34] Aviagen (2022) Ross 308 Broiler: Performance Objectives. Newbridge, Midlothian, Scotland, UK: Aviagen Limited.

[35] PIC (2019) Wean to Finish Guidelines. Nantwich, Cheshire, UK: PIC UK.

[36] Vukmirović D , Čolović R , Rakita S , et al. (2017) Importance of feed structure (particle size) and feed form (mash *v.* pellets) in pig nutrition – a review. Anim Feed Sci Tech 233, 133–144.

[37] Amerah AM , Ravindran V , Lentle RG , et al. (2007) Feed particle size: implications on the digestion and performance of poultry. Worlds Poult Sci J 63, 439–455.

[38] Chewning CG , Stark CR & Brake J (2012) Effects of particle size and feed form on broiler performance. J Appl Poult Res 21, 830–837.

[39] Branton SL , Lott BD , Morgan GW , et al. (1988) Research note: position of Meckel’s diverticulum in broiler-type chickens. Poult Sci 67, 677–679. 3405947 10.3382/ps.0670677

[40] Laerke H & Hedemann MS (2012) The digestive system of the pig. In Nutritional Physiology of Pigs, 1st ed., pp. 1–27 [ KE Bach Knudsen , et al., editors]. Foulum, Denmark: Videncenter for Svineproduktion.

[41] Al Masri S , Hüningen H & Aiyan A (2015) Influence of age at weaning and feeding regimes on the postnatal morphology of the porcine small intestine. J Swine Health Prod 23, 186–203.

[42] Bach Knudsen KE , Lærke HN , Steenfeldt S , et al. (2006) *In vivo* methods to study the digestion of starch in pigs and poultry. Anim Feed Sci Tech 130, 114–135.

[43] Lumpkins BS , Batal AB & Lee MD (2010) Evaluation of the bacterial community and intestinal development of different genetic lines of chickens. Poult Sci 89, 1614–1621. 20634515 10.3382/ps.2010-00747

[44] Lee SA , Dunne J , Mottram T , et al. (2017) Effect of diet phase change, dietary Ca and P level and phytase on bird performance and real-time gizzard pH measurements. Br Poult Sci 58, 290–297. 28277796 10.1080/00071668.2017.1293799

[45] Zheng L , Duarte ME & Sevarolli LA (2021) Intestinal health of pigs upon weaning: challenges and nutritional intervention. Front Vet Sci 8, 628258. 33644153 10.3389/fvets.2021.628258 PMC7906973

[46] Shewry PR & Halford NG (2002) Cereal seed storage proteins: structures, properties and role in grain utilization. J Exp Bot 53, 947–958. 11912237 10.1093/jexbot/53.370.947

[47] Hetland H , Svihus B & Olaisen V (2002) Effect of feeding whole cereals on performance, starch digestibility and duodenal particle size distribution in broiler chickens. Br Poult Sci 43, 416–423. 12195801 10.1080/00071660120103693

[48] Moran ET (editor) (1982) Comparative Nutrition of Fowl and Swine: The Gastrointestinal Systems, 1st ed. Guelph, Ontario, Canada: University of Guelph.

[49] Gao Q , Zhao F , Dang F , et al. (2020) Effect of corn particle size on the particle size of intestinal digesta or feces and nutrient digestibility of corn–soybean meal diets for growing pigs. Animals 10, 876. 32443473 10.3390/ani10050876 PMC7278416

[50] Park CS , Naranjo VD , Htoo JK , et al. (2020) Comparative amino acid digestibility between broiler chickens and pigs fed different poultry by-products and meat and bone meal. J Anim Sci 98, 1–8. 10.1093/jas/skaa223 PMC7455265 32667675

[51] Park CS , Helmbrecht A , Htoo JK , et al. (2017) Comparison of amino acid digestibility in full-fat soybean, two soybean meals, and peanut flour between broiler chickens and growing pigs. J Anim Sci 95, 3110–3119. 28727082 10.2527/jas.2017.1404

[52] Adedokun SA & Adeola O (2020) Regression-derived ileal endogenous amino acid losses in broiler chickens and cannulated pigs fed corn fibre, wheat bran, and pectin. Animals 10, 1–13. 10.3390/ani10112145 PMC7698621 33218020

[53] Takahashi T , Goto M & Sakata T (2004) Viscoelastic properties of the small intestinal and caecal contents of the chicken. Br J Nutr 91, 867–872. 15182390 10.1079/BJN20041129

[54] Takahashi T & Sakata T (2004) Viscous properties of pig cecal contents and the contribution of solid particles to viscosity. Nutrition 20, 377–382. 15043855 10.1016/j.nut.2003.12.011

[55] Lentle RG & Janssen PWM (2008) Physical characteristics of digesta and their influence on flow and mixing in the mammalian intestine: a review. J Comp Physiol B 178, 673–690. 18401586 10.1007/s00360-008-0264-x

[56] Choct M , Kocher A , Waters DLE , et al. (2004) A comparison of three xylanases on the nutritive value of two wheats for broiler chickens. Br J Nutr 92, 53–61. 15230987 10.1079/BJN20041166

[57] Passos AA , Park I , Ferket P , et al. (2015) Effect of dietary supplementation of xylanase on apparent ileal digestibility of nutrients, viscosity of digesta, and intestinal morphology of growing pigs fed corn and soybean meal based diet. Anim Nutr 1, 19–23. 29766982 10.1016/j.aninu.2015.02.006 PMC5884468

[58] Bedford MR & Schulze H (1998) Exogenous enzymes for pigs and poultry. Nutr Res Rev 11, 91–114. 19087461 10.1079/NRR19980007

[59] Jiang Z , Zhou Y , Lu F , et al. (2008) Effects of different levels of supplementary *α*-amylase on digestive enzyme activities and pancreatic amylase mRNA expression of young broilers. Asian-Australas J Anim Sci 21, 97–102.

[60] Pierzynowski S , Szwiec K , Valverde Piedra JL , et al. (2012) Exogenous pancreatic-like enzymes are recovered in the gut and improve growth of exocrine pancreatic insufficient pigs. J Anim Sci 90, 324–326. 23365368 10.2527/jas.53872

[61] Makkink CA & Verstegen MWA (1990) Pancreatic secretion in pigs. J Anim Physiol Anim Nutr (Berl) 64, 190–208.

[62] Osman AM (1982) Amylase in chicken intestine and pancreas. J Comp Physiol B 73, 571–574. 10.1016/0305-0491(82)90076-1 6185268

[63] Martens BMJ , Bruininx EMAM , Gerrits WJJ , et al. (2020) The importance of amylase action in the porcine stomach to starch digestion kinetics. Anim Feed Sci Tech 267, 114546.

[64] Liu X , Lyu W , Liu L , et al. (2021) Comparison of digestive enzyme activities and expression of small intestinal transporter genes in Jinhua and Landrace pigs. Front Physiol 12, 1–9. 10.3389/fphys.2021.669238 PMC8236719 34194337

[65] Doppenberg J & Van der Aar PJ (editors) (2017) Facts About Fats: A Review of the Feeding Value of Fats and Oils in Feeds for Swine and Poultry, 1st ed. Wageningen, Netherlands: Wageningen Academic Publishers.

[66] Ravindran V , Tancharoenrat P , Zaefarian F , et al. (2016) Fats in poultry nutrition: digestive physiology and factors influencing their utilisation. Anim Feed Sci Tech 213, 1–21.

[67] Tancharoenrat P , Ravindran V , Zaefarian F , et al. (2013) Influence of age on the apparent metabolisable energy and total tract apparent fat digestibility of different fat sources for broiler chickens. Anim Feed Sci Tech 186, 186–192.

[68] Wang S , Chao C , Cai J , et al. (2020) Starch–lipid and starch–lipid–protein complexes: a comprehensive review. Compr Rev Food Sci Food Saf 19, 1056–1079. 33331685 10.1111/1541-4337.12550

[69] Sano R , Shinozaki Y & Ohta T (2020) Sodium–glucose cotransporters: functional properties and pharmaceutical potential. J Diabetes Investig 11, 770–782. 10.1111/jdi.13255 PMC7378437 32196987

[70] Röder PV , Geillinger KE , Zietek TS , et al. (2014) The role of SGLT1 and GLUT2 in intestinal glucose transport and sensing. PLoS One 9, e89977. 24587162 10.1371/journal.pone.0089977 PMC3935955

[71] Byers MS , Howard C & Wang X (2017) Avian and mammalian facilitative glucose transporters. Microarrays 6, 1–15. 10.3390/microarrays6020007 PMC5487954 28379195

